# CBPDdb: a curated database of compounds derived from Coumarin–Benzothiazole–Pyrazole

**DOI:** 10.1093/database/baad062

**Published:** 2023-09-13

**Authors:** Shailima Rampogu, Mohammed Rafi Shaik, Merajuddin Khan, Mujeeb Khan, Tae Hwan Oh, Baji Shaik

**Affiliations:** Cachet Big Data Lab, Hyderabad, Telangana 500045, India; Department of Chemistry, College of Science, King Saud University, P.O. Box 2455, Riyadh 11451, Saudi Arabia; Department of Chemistry, College of Science, King Saud University, P.O. Box 2455, Riyadh 11451, Saudi Arabia; Department of Chemistry, College of Science, King Saud University, P.O. Box 2455, Riyadh 11451, Saudi Arabia; School of Chemical Engineering, Yeungnam University, Gyeongsan 38541, Republic of Korea; School of Chemical Engineering, Yeungnam University, Gyeongsan 38541, Republic of Korea

## Abstract

The present article describes the building of a small-molecule web server, CBPDdb, employing R-shiny. For the generation of the web server, three compounds were chosen, namely coumarin, benzothiazole and pyrazole, and their derivatives were curated from the literature. The two-dimensional (2D) structures were drawn using ChemDraw, and the .sdf file was created employing Discovery Studio Visualizer v2017. These compounds were read on the R-shiny app using ChemmineR, and the dataframe consisting of a total of 1146 compounds was generated and manipulated employing the dplyr package. The web server is provided with JSME 2D sketcher. The descriptors of the compounds are obtained using propOB with a filter. The users can download the filtered data in the .csv and .sdf formats, and the entire dataset of a compound can be downloaded in .sdf format. This web server facilitates the researchers to screen plausible inhibitors for different diseases. Additionally, the method used in building the web server can be adapted for developing other small-molecule databases (web servers) in RStudio.

**Database URL:** https://srampogu.shinyapps.io/CBPDdb_Revised/

## Introduction

Computer-aided drug design (CADD) has been instrumental in retrieving plausible inhibitors for a given target for the past three decades (1). This method allows quick screening of compounds at a very low cost (2–4). This CADD is accomplished either by structure-based drug design (SBDD) (5) or by ligand-based drug design (LBDD) (4).

In the SBDD, the presence of the resolved three-dimensional (3D) structure and its inbound ligand (small molecule) plays an important role (5). The interactions between the target and the ligand are critical in understanding the probable binding mode (6, 7) and important residues that might bring out the biological activity. An approach that demonstrates the association between the structure of a compound and its physicochemical properties that determine the biological activity of a compound is called the LBDD (8). The selection of the potential inhibitors is done either by mapping the compounds to a pharmacophore model and molecular docking (9–12) or directly by molecular docking (13). This process can be termed as screening or virtual screening. In 1997, the term virtual screening was first used in the literature (14) and is ‘defined as a set of computational methods that analyses large databases or collections of compounds in order to identify potential hit candidates’ (15). Generally, the search for the compounds is performed using the chemical libraries (16–19). Usually, the compounds are additionally filtered based on their drug-like properties in order to find favour during the development process.

A detailed account of different web servers embedded with small molecules is given in a study (14), while another web server provides information on different natural compounds with anticancer activity (20). However, a database with compound derivatives of coumarin, benzothiazole and pyrazole has not yet been built. Therefore, in the current study, we have built a web server of Coumarin–Benzothiazole–Pyrazole Derivatives Database (CBPDdb), with derivatives of coumarin, benzothiazole and pyrazole that have demonstrated biological activity towards various diseases.

## Materials and methods

### Collection of the compounds

In this study, three compounds, namely coumarin, benzothiazole and pyrazole, were selected to search for derivatives in the literature. These compounds were specifically chosen as there are an increasing number of experiments available on the biological activities of these derivatives. These compounds have demonstrated varied biological activities and therapeutic applications. We aim to provide the researchers in the field of CADD with most of the compounds with biological activities that would help them discover novel compounds for different diseases.

Specifically, the compounds that have shown biological activity was selected. The derivatives were collected by giving ‘compound names and their derivatives’, ‘compound name + synthesis’, ‘compound name + biological activity’ as the key words in PubMed, NCBI (https://pubmed.ncbi.nlm.nih.gov/), Google Scholar and Google.

The polyphenolic compounds coumarin (2H-1-benzopyran-2-one) are a group of oxygenated, colourless, crystalline compounds. These compounds were initially isolated from *Dipteryx odorata* Willd. (Fabaceae) in 1820 by Vogel. This plant is commonly called *Coumarou* (21, 22). Structurally, this compound is made up of a fused benzene ring and α-pyrone ring (23).

Benzothiazole is a heterocyclic structure that is usually bioactive (24). These compounds have a heterocyclic nucleus called a thiazole that confers various biological properties (25). The π-excess aromatic heterocyclic compound pyrazole is a five-membered structure, which is a widely studied group in the azole family (26). The pyrazole template has gained popularity due to its potential therapeutic applications (26). In this compound, the fourth position is preferred for the electrophilic substitution reaction, while the third and the fifth positions are preferred by the nucleophilic reactions (26). To the pyrazole ring, several varied functional groups can be added, substituted, removed or fused to correspondingly synthesize the biologically potent compounds (27). These three compounds have various medicinal applications and hence are chosen to generate a web server with their derivatives (25, 28–33).

### Building of the webserver

**Figure 1.**
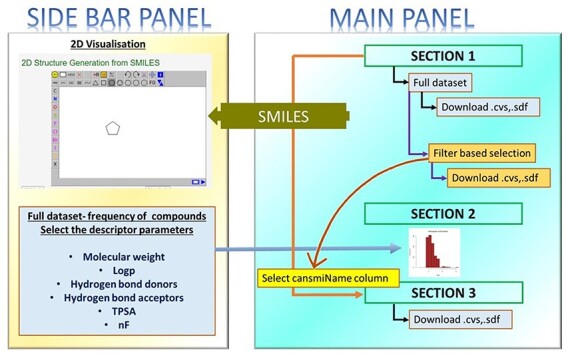
Overview of the web server.

The two-dimensional (2D) structures were initially sketched employing ChemDraw and saved in .mol format. These structures were upgraded to Discovery Studio Visualizer to obtain their 3D forms and saved them in .sdf format. The therapeutic action of the compounds and the source of curation were prepared in a .csv file that was used to develop the server along with the .sdf files of the compounds. The overview of the web server is given in Figure 1.

To build the web server, the ChemmineR (34) was used that enables compound similarity search, clustering, visualization and function of compounds. Here, we have employed the DT (renderDataTable) to display the data of the compounds into a data table form.

## Results

### Collection of the compounds and building of CBPDdb

For building a web server that could help the computational chemist, computational biologist or CADD researchers, we have selected coumarins, benzothiazole and pyrazole as a first attempt. A total of 1146 compounds (coumarin, 140; benzothiazole, 451 and pyrazole, 555) were curated from various literature sources. Using the read.SDFset available with ChemmineR, the compounds were imported into the RStudio. The properties/descriptors for these compounds were generated employing propOB. This feature can be adapted post instalment of ChemmineOB package and the OpenBabel software (35). The so-obtained results are transformed into a data table (DT1).

Furthermore, a different file was generated in .csv format that included the therapeutic action and source of data curation. This file was also read on RStudio using read.csv and a data table (DT2) was created. The two data tables (DT1 and DT2) were merged to join the descriptors with the therapeutic action using the merge function and dplyr. This final data table was displayed on the web server. This pattern was followed to generate the data table for the derivatives, which were displayed under three tabs.

### How to use the database

The web server is divided into three major sections: (1) full dataset with filters, (2) full dataset graphical frequency analysis of descriptors and (3) extracting cansmi (smiles) column: filtered data.

#### Full dataset with filters

This section shows the full dataset of the compounds. The derivatives of the three compounds are included in a separate tab that can be downloaded in the .csv or .sdf formats. Each of the data tables is provided with a top filter that allows the users to choose their choice of descriptors. The filtered data can be downloaded as a .csv file and checked if the selected compounds are downloaded by counter-verifying the Chemical Name in both the files (Supplementary Figure 1). The DT is equipped with clickable links that correspondingly connects to the compound articles. The DT is provided with a search bar that allows the users to search a given input. For instance, if anticancer is given as an input, the results in the DT will display only those compounds with anticancer property.

#### Full dataset graphical frequency analysis of descriptors

The sidebar panel of the web server is equipped with a histogram plot that displays the frequency of the compounds. The users can select the descriptor from the sidebar panel and view the result as a histogram with the selection option for bins (Supplementary Figure 2).

#### Extracting the cansmi (smiles) column: filtered data

Section 3 is linked to Section 1, which specifically retrieves a single column upon selection. Once the data is filtered (Section 1), the cansmiName column is selected in Section 1. The selected column with the filtered data will be displayed in Section 3. Here, the display corresponds to the selected tab. The results (filtered data) can be downloaded in the .csv and .sdf formats. The .sdf files can be used to generate the 3D structures (Supplementary Figure 3).

## Visualizing the 2D structures

The sidebar panel of the server is embedded with JSME Molecular Editor (Supplementary Figure 2) (36), which facilitates the visualization of the structure of the compounds. The 2D structures can be viewed by giving the SMILES (cansmi, which are the Canonical SMILES) as an input at the Molecular Editor by clicking the downward arrow, selecting the Paste Mol or SDF or SMILES and clicking Accept. The 2D structure appears on the editor (Supplementary Figure 4). The editor also has other parameters through which the compound’s appearance can be changed. Additionally, the users can copy and save the compound in several formats. The modification of the molecules is supported by JSME by clicking the FG (36) (Supplementary Figure 5).

## Discussion and conclusion

In order to discover new drugs with therapeutic ability, the CADD process plays a very effective role. In contrast, traditional drug discovery methods are time- and money-consuming processes (2). The term CADD includes saving the compounds, organizing and evaluating them and further modelling the compounds (2). The efficiency of CADD can be evidently seen during the recent pandemic times, when there was an urgency to identify the potential candidate compounds (37–39). Earlier, our group had computationally designed butein analogues that demonstrated anticancer activity (40). Furthermore, these compounds have shown in silico antibacterial activity (41). In another study, computational design of PARP inhibitors was performed against SARS-CoV-2 (42).

Virtual screening is an important step in retrieving the best molecule against a given target (43, 44). The screening process may proceed via SBDD and/or LBDD (43). In either methods, the main purpose is to discover a highly potent putative compound against a target (44, 45). The molecular docking is also included in the virtual screening step. Molecular docking primarily imparts knowledge on the binding mode of the ligand at the active site of the protein (46).The small molecules can be prepared using Gypsum-DL for structure-based virtual screening (47).

Accordingly, in the present study, we have built a web server called the CBPDdb, consisting of derivatives of compounds from coumarin, benzothiazole and pyrazole curated from different literature sources. These compounds have displayed biological activities such as anticancer, antifungal, antiviral, etc. We believe that these compounds will be useful for the CADD researchers to work with the compounds for using them against several diseases. This web server is equipped with JSME, a 2D sketcher that enables the users to visualize the 2D structures of the compounds. Furthermore, the compounds can be selected based on filter parameters to facilitate the user’s choice of compounds.

In the following versions, the web server will be regularly updated to increase the number of compounds with the coumarin, benzothiazole and pyrazole derivatives and other derivatives. Furthermore, the web server will be incorporated with different analysis methods and predictions relevant to medicinal chemistry and CADD.

In conclusion, we believe that this web server could help the computational chemist or computational biologist in their research progress. Furthermore, our attempt may also help the researchers design new small-molecule web servers.

## Supplementary Material

baad062_Supp

## Data Availability

The data underlying this article are available in https://github.com/SRampogu/CBPDdb_revised.
