# Supplementary material for: CBPDdb: a curated database of compounds derived from Coumarin–Benzothiazole–Pyrazole
Source: Database (Oxford). 2023 Sep 13;2023:baad062. doi: 10.1093/database/baad062 (PMC10498939; doi:10.1093/database/baad062)
Supplement: baad062_Supp [file baad062_supp.zip › Supplementary.pdf]

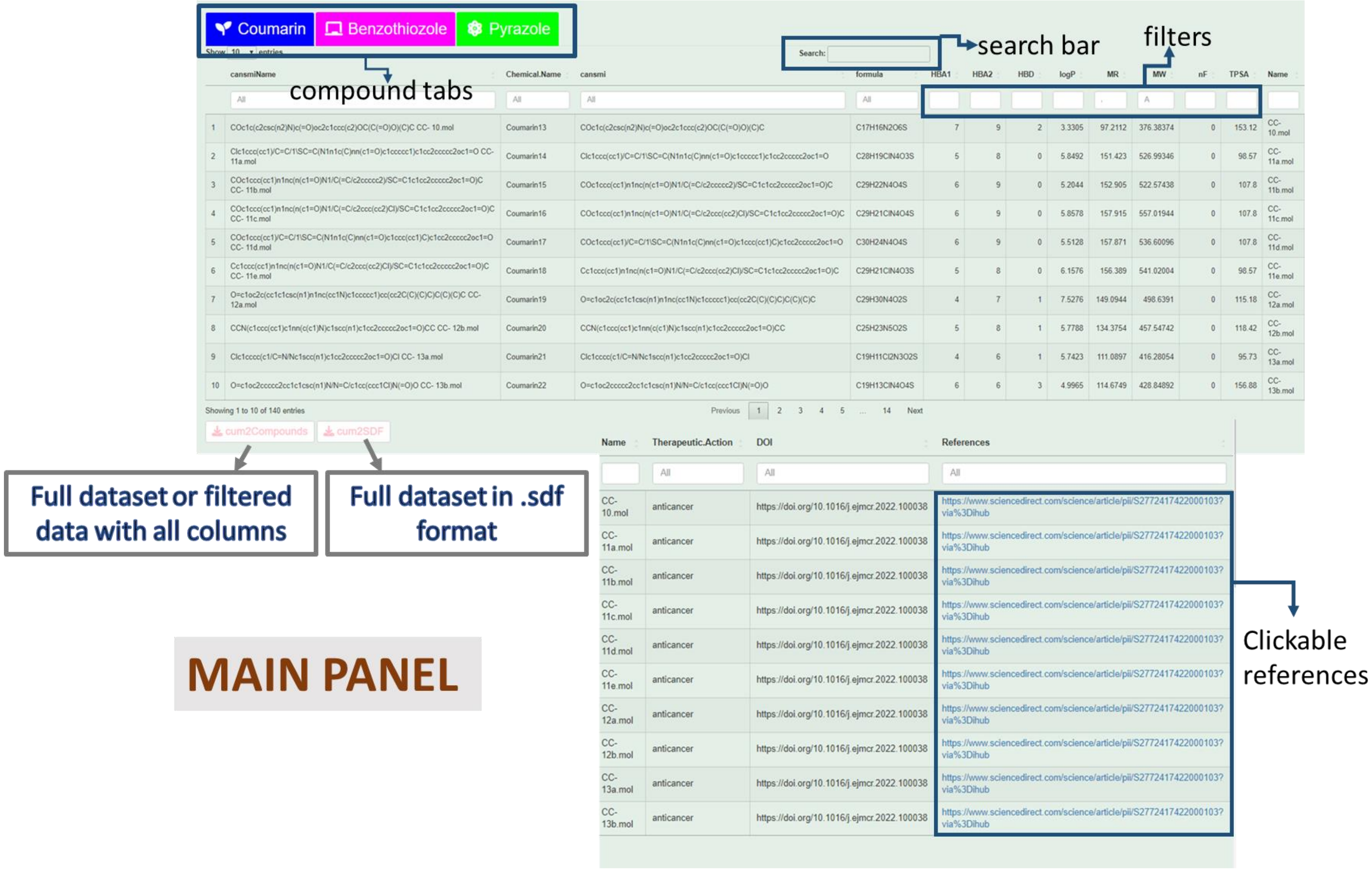

Supplementary Figure 1. The main panel with the available filters.

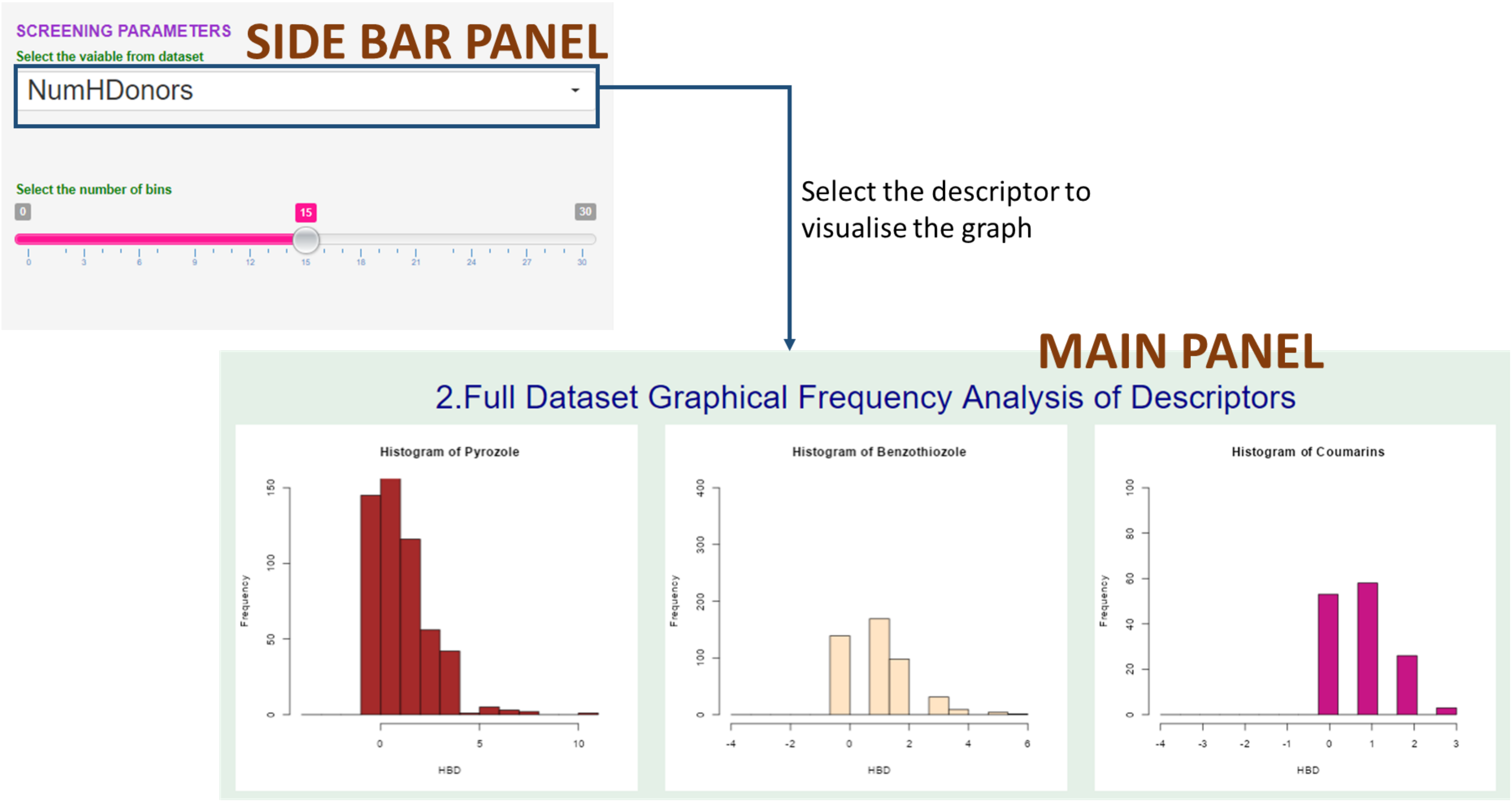

Supplementary Figure 2. Frequency of graphical visualization of the compounds corresponding to the descriptors

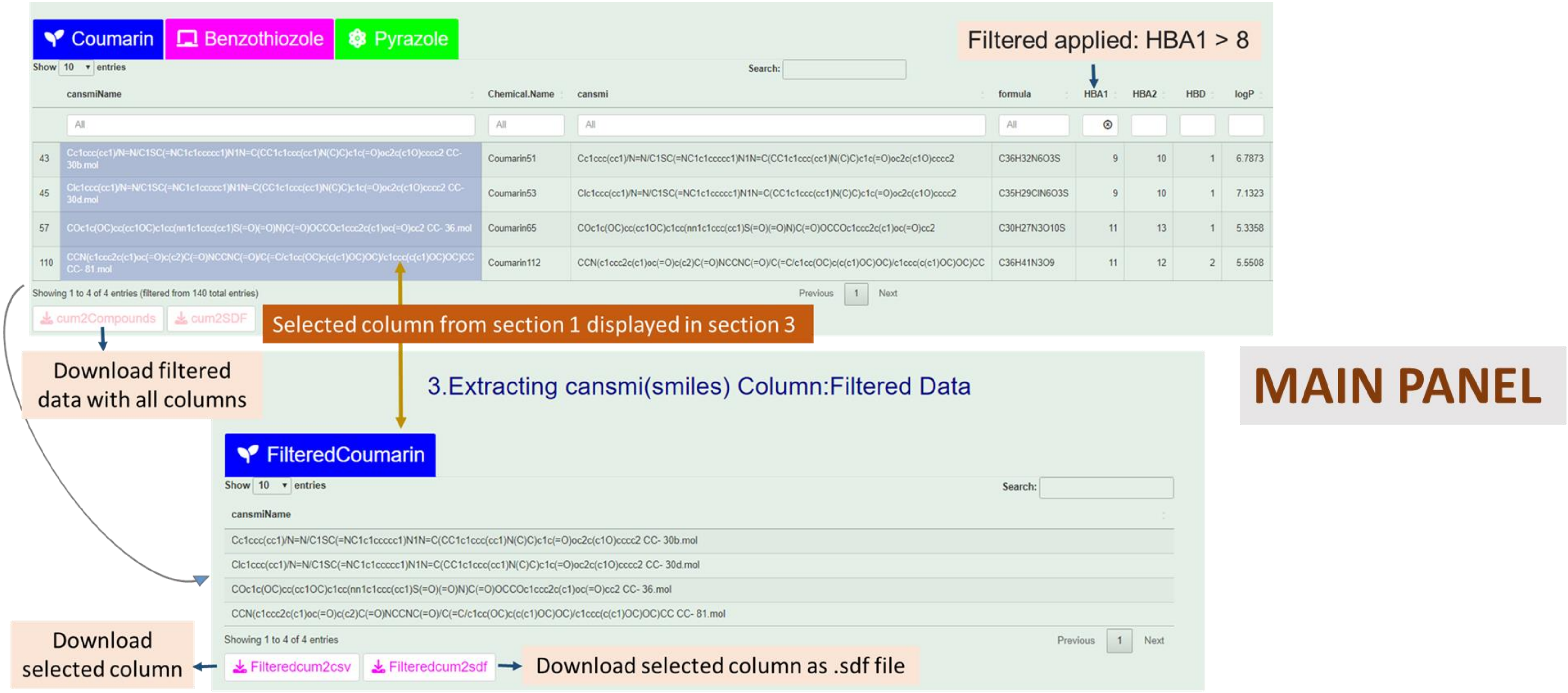

Supplementary Figure 3. Filter-based column selection and download the filtered data as .csv and .sdf files.

SIDE BAR PANEL

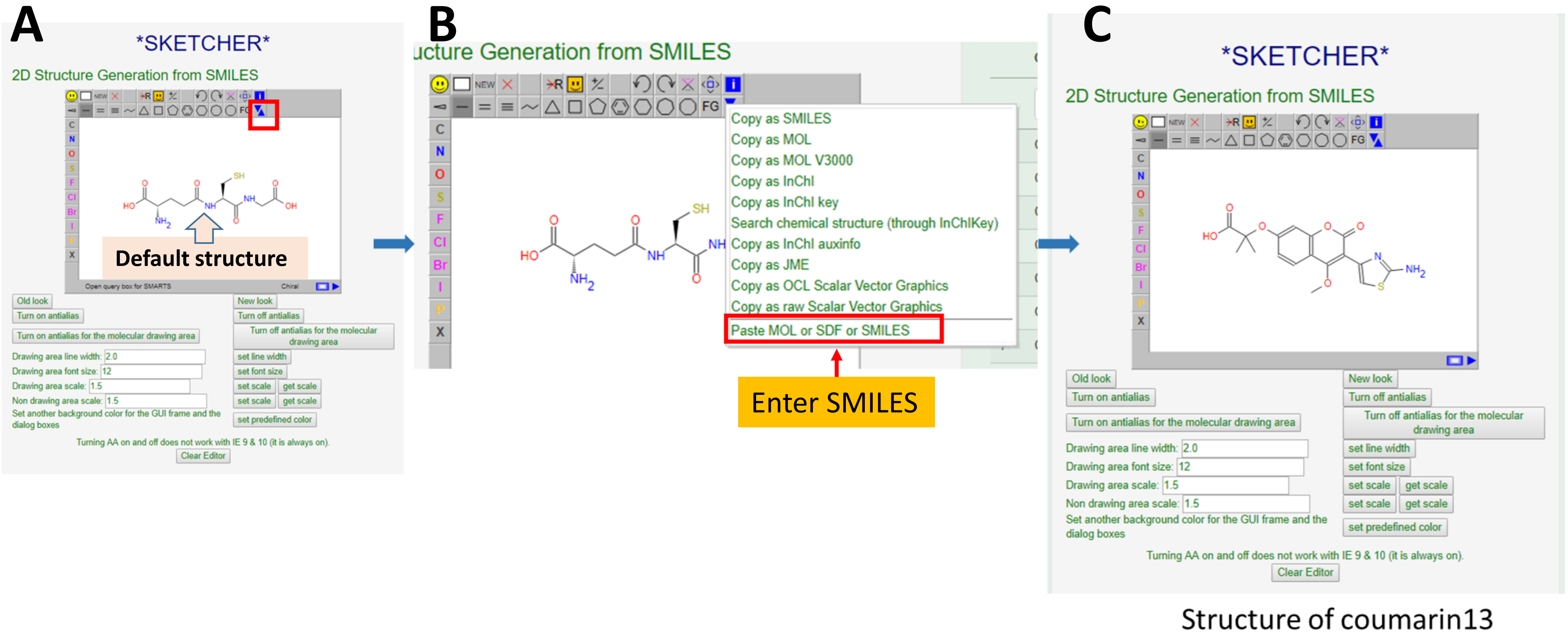

Supplementary Figure 4. The step-wise process of viewing the 2D structures of the compounds giving input as SMILES.

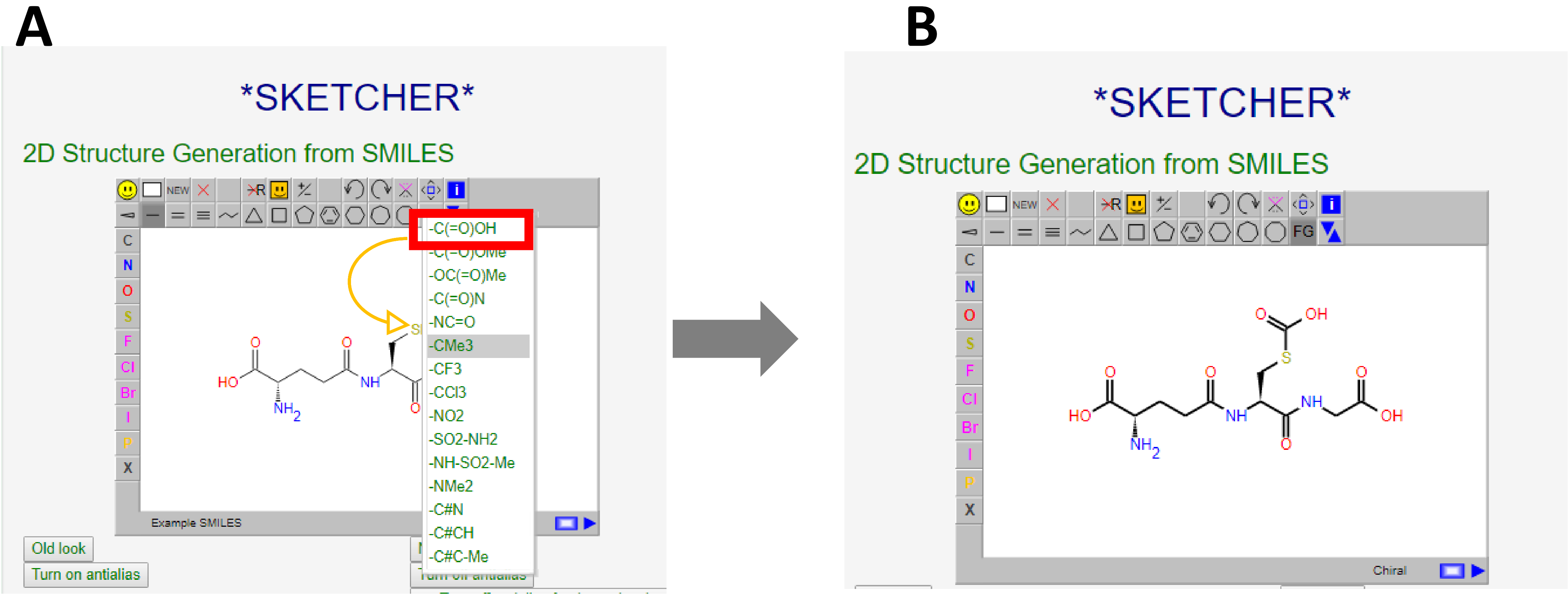

Supplementary Figure 5. Editing a given molecule.
